# Peritumoral plasmacytoid dendritic cells predict a poor prognosis for intrahepatic cholangiocarcinoma after curative resection

**DOI:** 10.1186/s12935-020-01676-z

**Published:** 2020-12-04

**Authors:** Zhi-Qiang Hu, Zheng-Jun Zhou, Chu-Bin Luo, Hao-Yang Xin, Jia Li, Song-Yang Yu, Shao-Lai Zhou

**Affiliations:** 1 Liver Surgery Department, Liver Cancer Institute, Zhongshan Hospital, Fudan University, 136 Yi Xue Yuan Road, Shanghai, 200032 China; 2 Key Laboratory of Carcinogenesis and Cancer Invasion (Fudan University), Ministry of Education, Shanghai, 200032 China

**Keywords:** Plasmacytoid dendritic cells, Intrahepatic cholangiocarcinoma, Treg cells, Prognosis, Recurrence

## Abstract

**Background:**

Plasmacytoid dendritic cells (pDCs) are present in various primary and metastatic human neoplasms; however, their clinical significance in intrahepatic cholangiocarcinoma is not clear.

**Methods:**

To evaluate pDCs’ distributions in and around tumors as well as their potential function and predictive value for prognosis in patients undergoing curative resection, we performed immunohistochemistry to examine the expression of pDC marker BDCA2, and CD3, CD4, CD8 and Foxp3 in intratumoral and peritumoral tissues from 359 patients with intrahepatic cholangiocarcinoma and compared with prognostic and clinicopathologic factors.

**Results:**

Results showed that patients with high numbers of BDCA2^+^ pDCs in peritumoral tissues were more likely to have elevated levels of carbohydrate antigen 19-9 and gamma-glutamyl transferase, larger and more tumors, advanced tumor-node-metastasis staging, more vascular/bile duct invasion, and lymphatic metastasis in association with greater chance of recurrence and shorter overall survival. Peritumoral tissues with larger numbers of pDCs also showed increased Foxp3^+^ regulatory T cell infiltration, both of which were found to be independent factors for predicting time to recurrence and overall survival. By contrast, patient outcomes were not associated with the presence of intratumoral pDCs.

**Conclusions:**

Peritumoral pDC infiltration may indicate an immune tolerogenic peritumor microenvironment and can be used to predict a poor prognosis for patients undergoing curative resection for intrahepatic cholangiocarcinoma.

**Electronic supplementary material:**

The online version of this article (10.1186/s12935-020-01676-z) contains supplementary material, which is available to authorized users.

## Introduction

Intrahepatic cholangiocarcinoma (ICC) is one of most common types of cancer in the liver, second only to hepatocellular carcinoma, and the incidence of ICC continues to increase worldwide [1, 2]. Curative resection for ICC relies on early detection, such as by clinical screenings with advanced diagnostic techniques. Nevertheless, the high rate of ICC recurrence shortens the long-term survival of patients [3, 4]. To better predict recurrence in patients after curative resection and enable more effective treatments, the molecular mechanisms contributing to disease recurrence and progression need to be elucidated.

The types and densities of immune and nonimmune stromal cells as well as their locations within the tumor microenvironment have been identified as prognostic factors for several types of cancer [5–7]. Of note, ICC typically exhibits a prominent stromal reaction involving tissue-associated macrophages, tumor-associated neutrophils, and various types of T lymphocytes [8]. Immune/stromal cells are increasingly implicated in controlling invasive tumor growth and metastasis, resistance to chemotherapies and targeted agent therapies, and immunosuppression in patients with ICC [9].

Plasmacytoid dendritic cells (pDCs), which generate large amounts of type I interferon, represent a first line of defense against infection [10]. There is evidence that pDCs regulate T cell-mediated adaptive and innate immunity and thus likely contribute to cancer immunity [11, 12]. pDCs are responsible for creating an immunosuppressive microenvironment in a variety of tumors [13]. For example, the immune tolerance induced by pDCs is crucial for the progression of ovarian cancers, and high numbers of pDCs in breast tumors are associated with dissemination and relapse [14, 15]. Notably, the growth of tumors and their metastasis to bone tissues are suppressed when pDCs are depleted [16]. pDCs also influence the progression of multiple myeloma by promoting the survival and growth of tumors and contributing to chemotaxis and drug resistance [17].

Although pDCs have been observed in various tumor types, their distribution within these tumors and their potential interactions with other cells are largely unexplored [13]. To address this, we characterized the distribution of pDCs in ICC and determined if they can be associated with patient outcomes. Furthermore, we investigated the potential mechanism (s) by which pDCs regulate the immune microenvironment in ICC tumors.

## Materials and methods

### Patients

This study included 359 patients with ICC that underwent curative resection at Zhongshan Hospital, Fudan University, between 2009 and 2013. Patients were excluded from the analysis if they had palliative surgery or prior intervention (such as transhepatic artery embolization and chemo- or radiotherapy). Patients who developed another type of primary malignancy or inflammatory disease during the follow-up were also excluded. Informed consent was obtained from all participants, and the study was approved by the Research Ethics Committee of Zhongshan Hospital. Detailed information is provided in Table 1.

**Table 1:** Clinicopathologic characteristics of patients with intrahepatic cholangiocarcinoma (n = 359)

Characteristics	Number (%)
Age, year (≤ 50 versus > 50)	74/285 (20.6/79.4)
Sex (female versus male)	153/206 (42.6/57.4)
HBsAg (negative versus positive)	252/107 (70.2/29.8)
AFP, ng/ml (≤ 20 versus > 20)	317/42 (88.3/11.7)
CA199 (≤ 36 versus > 36)	149/210 (41.5/58.5)
GGT,U/L (≤ 54 versus > 54)	172/187 (47.9/52.1)
Liver cirrhosis (no versus yes)	273/86 (76.0/24.0)
Tumor size, cm (≤ 5 versus > 5)	159/200 (44.3/55.7)
Tumor number (single versus multiple)	258/101 (71.9/28.1)
Microvascular/bile duct invasion (no versus yes)	282/77 (78.6/21.4)
Lymphatic metastasis (no versus yes)	306/53 (85.2/14.8)
Tumor encapsulation (complete versus none)	62/297 (17.3/82.7)
Tumor differentiation (I + II versus III + IV)	181/178 (50.4/49.6)
TNM stage (I versus II + III + IV)	182/177 (50.7/49.3)

*AFP* alpha-fetoprotein, *GGT* gamma glutamyl transferase, *CA 19-9* carbohydrate antigen 19-9, *TNM* tumor-node-metastasis

### Diagnostic criteria

Diagnoses from histopathology were in accordance with World Health Organization criteria. Tumor differentiation was graded according to Edmondson and Steiner [18], and tumor staging was determined via tumor-node-metastasis (TNM) grading according to the 2017 guidelines of the International Union Against Cancer. Liver functioning was assessed via Child–Pugh scoring [19].

### Clinical outcomes

Patients were monitored after the procedure as described previously [20]. Overall survival (OS) was defined as the time from the surgery until death or the final follow-up, when data from surviving patients were censored, in December of 2018. Time to recurrence (TTR) was defined as the time from the surgery until intrahepatic recurrence or extrahepatic metastasis was diagnosed [21].

### Immunohistochemical analyses

Immunohistochemistry was performed on tissue microarrays comprising two 2-mm-diameter biopsy samples (spots) of 359 intratumoral and 322 peritumoral tissues. The absence of necrotic or hemorrhagic damage was confirmed by hematoxylin and eosin staining.

After antigen retrieval, tissue sections were incubated overnight at 4 °C with the following antibodies: anti-human blood dendritic cell antigen 2 (anti-BDCA2) (1:200; Abnova), anti-CD3 (clone F7.2.38) and anti-CD8 (clone C8/144B) (1:50; DakoCytomation), anti-CD4 (clone EPR6855) (1:100; Epitomics), and Foxp3 (clone 236A/E7) (1:100, Abcam). The sections were then incubated for 30 min at 37 °C with horseradish-peroxidase secondary antibodies from the EnVision Detection kit (GK500705, Gene Tech, China) and visualized by reacting with 3,3′-diaminobenzidine after avidin–biotin-mediated amplification, as described previously [22]. Mayer’s hematoxylin was used for counterstaining. Omission of the primary antibody served as a negative control.

The numbers of immunopositive cells in each tissue spot were quantified by three investigators blinded to the sample identification and expressed as mean number (± standard deviation) of cells/spot of the triplicate results. In subsequent analyses, the medians were used as the cutoff values unless specified otherwise. Thus, for BDCA + pDCs, those with ≤ 28 cells/spot were assigned to the pDCs^low^ group, and those with > 28 cells/spot were assigned to the pDCs^high^ group. For Foxp3 + T regulatory (Treg) cells, those with ≤ 7 cells/spot were assigned to the Tregs^low^ group, and those with > 7 cells/spot were assigned to the Tregs^high^ group.

### Statistical analysis

Differences in the numbers of immunopositive cells between groups were assessed with unpaired-sample *t* tests. To evaluate associations between immunohistochemistry results and clinical characteristics, Chi square and Fisher’s exact tests were performed. Correlations were evaluated by means of Spearman’s rho coefficients. OS and cumulative recurrence rates were analyzed with the Kaplan–Meier method and log-rank test. Univariate and multivariate analyses were performed via Cox proportional hazards regression. All analyses were performed with SPSS 16.0 statistical software. A *p* value of < 0.05 was considered statistically significant.

## Results

### Peritumoral pDC abundance in ICC patients correlates with clinicopathologic features

**Fig. 1:**
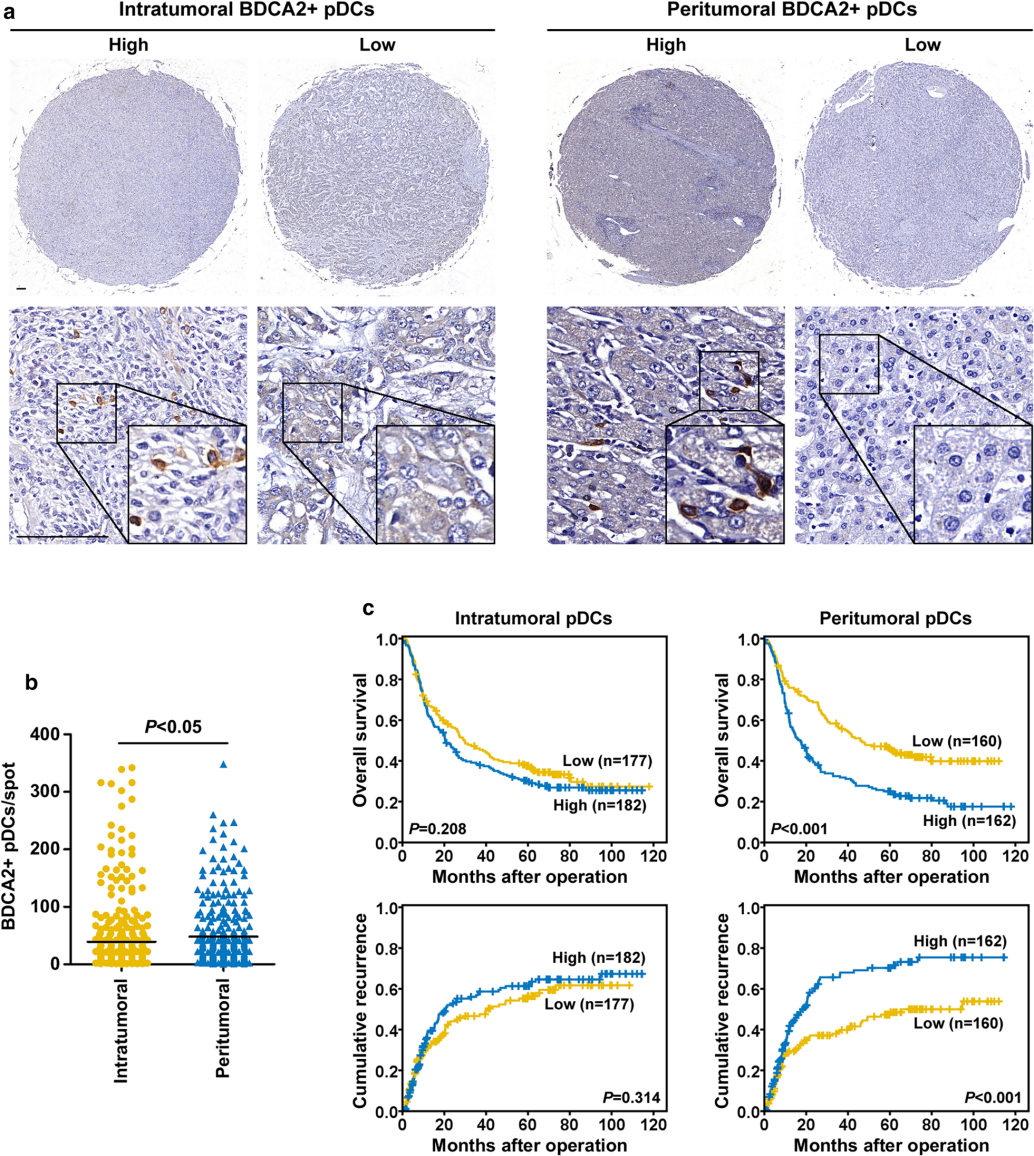
pDC distribution in biopsy samples and the association with ICC prognosis. **a** Representative ICC tumor samples showing BDCA2^+^ pDCs in tumoral and peritumoral tissues. Scale bar, 100 μm. **b** pDCs were more abundant in peritumoral tissues than in intratumoral areas. **c** Prognostic value of pDCs in tumoral and peritumoral tissues of ICC patients

BDCA2-immunopositive pDCs were more abundant in peritumoral tissue than in intratumoral areas (48.3 ± 55.4 cells/spot vs. 39.3 ± 62.8 cells/spot, respectively; *p *< 0.05) (Fig. 1a, b). The number of peritumoral pDCs was significantly positively correlated with carbohydrate antigen 19-9 and gamma-glutamyl transferase levels (*p *= 0.025 and *p *= 0.01, respectively), tumor size (*p *= 0.007), tumor number (*p *= 0.043), degrees of vascular/bile duct invasion and lymphatic metastasis (*p *= 0.045 and *p *= 0.018, respectively), and TNM stage (*p *= 0.002) (Table 2). By contrast, sex was the only clinical characteristic that correlated with the number of pDCs in intratumoral tissues.

**Table 2:** Correlation between intratumoral and peritumoral plasmacytoid dendritic cells (pDCs) and clinicopathologic characteristics in ICC (n = 359 for intratumoral tissues, 322 for peritumoral tissues)

Clinicopathological indexes	Intratumoral pDCs			Peritumoral pDCs		
	Low	High	P	Low	High	P
Age (year)						
≤ 50	32	42	0.242	32	36	0.625
> 50	145	140		128	126	
Sex						
Female	85	68	*0.041*	75	61	0.094
Male	92	114		85	101	
HBsAg						
Negative	124	128	0.955	110	116	0.576
Positive	53	54		50	46	
CA199						
≤ 36	78	71	0.331	78	59	*0.025*
> 36	99	111		82	103	
AFP (ng/ml)						
≤ 20	157	160	0.816	145	141	0.307
> 20	20	22		15	21	
GGT (U/L)						
≤ 54	80	92	0.310	89	67	*0.010*
> 54	97	90		71	95	
Liver cirrhosis						
No	138	135	0.400	120	127	0.471
Yes	39	47		40	35	
Tumor size (cm)						
≤ 5	74	85	0.351	84	61	*0.007*
> 5	103	97		76	101	
Tumor number						
Single	130	128	0.511	122	107	*0.043*
Multiple	47	54		38	55	
Vascular/bile duct invasion						
Absence	132	150	0.070	134	121	*0.045*
Present	45	32		26	41	
Lymphatic metastasis						
No	152	154	0.736	145	132	*0.018*
Yes	25	28		15	30	
Tumor encapsulation						
Complete	37	25	0.072	32	23	0.167
None	140	157		128	139	
Tumor differentiation						
I + II	93	88	0.427	90	79	0.179
III + IV	84	94		70	83	
TNM stage						
I	90	92	0.955	97	70	*0.002*
II + III + IV + IV	87	90		63	92	

The italic numerals indicate P value < 0.05

*AFP* alpha-fetoprotein, *GGT* gamma glutamyl transferase, *CA 19-9* carbohydrate antigen 19-9, *TNM* tumor-node-metastasis

Chi square tests for all analyses

### Peritumoral pDC accumulation in ICC patients is a predictor of poor prognosis

At the time of the final follow-up examination, 68.2% (245/359) of the patients had died, and 49.6% (178/359) had experienced recurrence. The 1-, 3-, and 5-year rates were 65.7%, 42.3%, and 33.8%, respectively, for OS and 34.8%, 51.6%, and 58.7% for cumulative recurrence.

We next classified patients according to the number of peritumoral pDCs: those with ≤ 28 cells/spot were assigned to the pDCs^low^ group, and those with > 28 cells/spot were assigned to the pDCs^high^ group. Patients in the pDCs^low^ group had significantly higher 1-, 3-, and 5-year OS rates than those in the pDCs^high^ group (75.9% versus 59.6%, 56.4% versus 32.4%, and 46.4% versus 24.9%, respectively) (Fig. 1c). Patients in the pDCs^high^ group also showed higher cumulative recurrence rates at 1, 3, and 5 years than those in the pDCs^low^ group (41.0% versus 29.0%, 64.7% versus 38.4%, and 69.5% versus 48.2%, respectively) (Fig. 1c). Additionally, peritumoral pDCs were associated with OS and cumulative recurrence rates in patients with early-stage (TNM stage I) ICC (*n *= 182) and normal carbohydrate antigen 19-9 levels (≤ 36 ng/ml, *n *= 149) (Additional file 1: Figure S1). Of note, OS and cumulative recurrence rates were not associated with the number of intratumoral pDCs (Fig. 1c).

In the univariate analysis, prolonged TTR and OS were associated with a lower number of peritumoral pDCs in addition to various clinicopathologic factors (Table 3). The multivariate analysis revealed that the abundance of peritumoral pDCs, along with tumor number, lymphatic metastasis, and tumor encapsulation, was an independent factor for OS (*p *= 0.002, hazard ratio [HR] = 1.55) and TTR (*p *=0.01, HR = 1.54) (Table 3).

**Table 3:** Univariate and multivariate analyses of prognostic factors in ICC (n = 359)

Variable	TTR		OS	
	HR (95% CI)	*P*	HR (95% CI)	P
Univariate analysis				
Age, year (≤ 50 versus > 50)	0.89 (0.63–1.28)	0.551	1.29 (0.94–1.78)	0.116
Sex (female versus male)	1.04 (0.77–1.39)	0.809	1.07 (0.83–1.38)	0.583
HBsAg (negative versus positive)	1.09 (0.80–1.50)	0.577	0.72 (0.54–0.96)	*0.025*
AFP, ng/ml (≤ 20 versus > 20)	1.17 (0.76–1.79)	0.483	0.84 (0.56–1.26)	0.396
CA199 (≤ 36 versus > 36)	1.19 (0.88–1.60)	0.258	1.90 (1.46–2.48)	*0*
GGT,U/L (≤ 54 versus > 54)	1.35 (1.00–1.81)	0.05	1.92 (1.48–2.48)	*0*
Liver cirrhosis (no versus yes)	1.16 (0.83–1.62)	0.396	0.83 (0.61–1.12)	0.226
Tumor size, cm (≤ 5 versus > 5)	1.38 (1.02–1.86)	*0.035*	1.65 (1.28–2.14)	*0*
Tumor number (single versus multiple)	2.48 (1.81–3.40)	*0*	2.66 (2.03–3.47)	*0*
Microvascular/bile duct invasion (no versus yes)	1.02 (0.70–1.48)	0.937	1.48 (1.10–1.97)	*0.009*
Lymphatic metastasis (no versus yes)	2.23 (1.52–3.28)	*0*	2.70 (1.96–3.72)	*0*
Tumor encapsulation (complete versus none)	1.91 (1.20–3.04)	*0.006*	1.46 (1.01–2.11)	*0.042*
Tumor differentiation (I + II versus III + IV)	1.10 (0.82–1.48)	0.519	1.43 (1.11–1.84)	*0.005*
TNM stage (I versus II + III + IV)	1.92 (1.42–2.59)	*0*	2.78 (2.14–3.61)	*0*
Intra-pDCs (low versus high)	1.16 (0.87–1.56)	0.314	1.18 (0.91–1.51)	0.208
Peri-pDCs (low versus high)	1.77 (1.29–2.44)	*0*	1.87 (1.42–2.46)	*0*
Peri-Tregs (low versus high)	1.63 (1.19–2.24)	*0.003*	1.53 (1.17–2.01)	*0*
Peri-pDCs and Tregs (both low vs. both high)	2.22 (1.49–3.31)	*0*	2.17 (1.55–3.03)	*0*
Multivariate analysis				
HBsAg (negative versus positive)	NA	NA	0.70 (0.51–0.97)	*0.033*
CA199 (≤ 36 versus > 36)	NA	NA	1.35 (1.01–1.82)	*0.045*
GGT, U/L (≤ 54 versus > 54)	NA	NA	1.65 (1.24–2.21)	*0.001*
Tumor size, cm (≤ 5 versus > 5)	1.05 (0.75–1.46)	0.786	1.13 (0.84–1.51)	0.435
Tumor number (single versus multiple)	1.94 (1.36–2.77)	*0*	2.12 (1.57–2.86)	*0*
Microvascular/bile duct invasion (no versus yes)	NA	NA	1.28 (0.91–1.79)	0.153
Lymphatic metastasis (no versus yes)	1.72 (1.12–2.64)	*0.014*	2.09 (1.45–3.00)	*0*
Tumor encapsulation (complete versus none)	1.96 (1.18–3.25)	*0.009*	1.58 (1.04–2.42)	*0.034*
Tumor differentiation (I + II versus III + IV)	NA	NA	1.32 (1.00–1.74)	0.051
Peri-pDCs (low versus high)	1.54 (1.11–2.14)	*0.01*	1.55 (1.17–2.05)	*0.002*
Peri-Tregs (low versus high)	1.59 (1.16–2.20)	*0.004*	1.34 (1.02–1.77)	*0.036*
Peri-pDCs and Tregs (both low vs. both high)	1.96 (1.30–2.94)	*0.001*	1.74 (1.23–2.47)	*0.002*

The italic numerals indicate P value < 0.05

Cox proportional hazards regression model

*AFP* alpha-fetoprotein, *GGT* gamma glutamyl transferase, *CA 19-9* carbohydrate antigen 19-9, *TNM* tumor-node-metastasis, *HR* hazard ratio, *CI* confidential interval

### pDC accumulation is associated with Treg abundance in peritumoral tissues

**Fig. 2:**
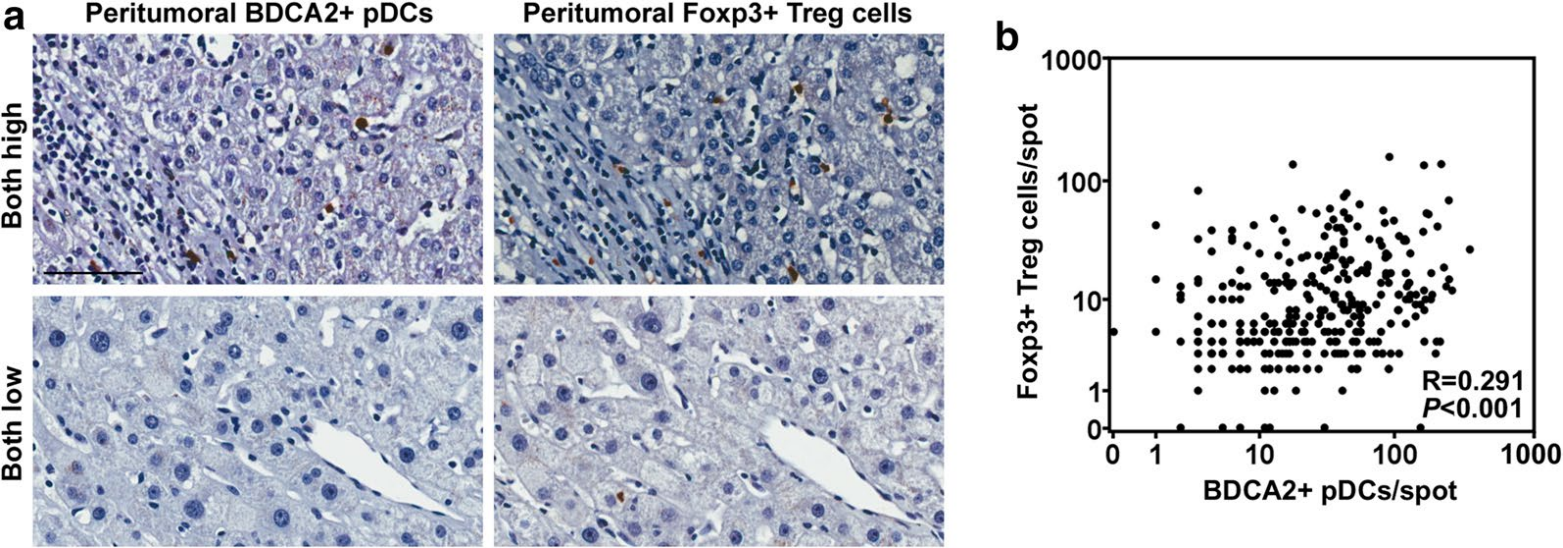
Correlation between peritumoral pDC and Treg abundance. **a** High and low infiltration of peritumoral pDCs and Tregs. Scale bar, 100 μm. **b** Scatterplots showing significant positive correlations between BDCA2^+^ pDCs and Tregs in peritumoral tissues

To examine the association between pDCs and T cell-mediated immune responses, T lymphocytes in peritumoral tissues were immunostained (Fig. 2a). The number of Foxp3^+^ Tregs was significantly positively correlated with the number of peritumoral pDCs (*p *< 0.001, *R *= 0.291) (Fig. 2b). However, the numbers of CD3^+^, CD4^+^, and CD8^+^ lymphocytes were not correlated (Additional file 2: Table S1).

### Combination of peritumoral pDC and Treg abundance for predicting ICC patient outcomes

**Fig. 3:**
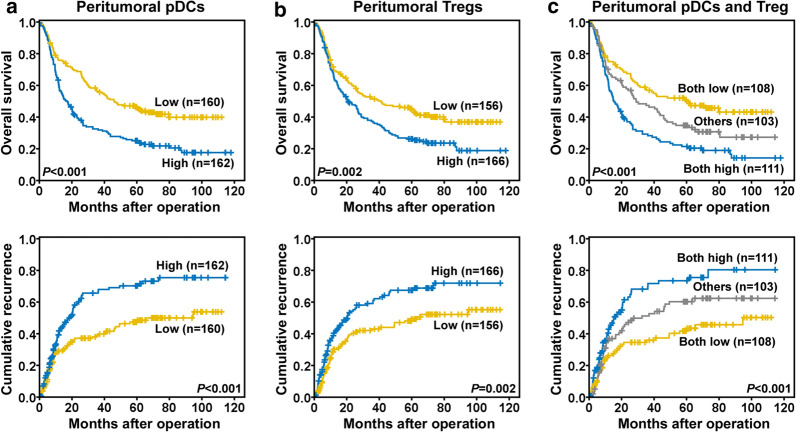
Prognostic values of peritumoral pDC and Treg accumulation in ICC patients. Prognostic value of pDCs (**a**), Tregs (**b**), and pDCs plus Tregs (**c**) in peritumoral tissues of ICC patients

As the numbers of pDCs and Tregs were correlated, we evaluated the prognostic value of these factors combined. We compared the prognoses of ICC patients categorized into three groups according to peritumoral cell abundance: pDCs^low^/Tregs^low^, pDCs^low^/Tregs^high^ or pDCs^high^/Tregs^low^, and pDCs^high^/Tregs^high^. The 1-, 3-, and 5-year OS rates for the patients in the pDCs^high^/Tregs^high^ group were 60.2%, 29.1%, and 21.3%, respectively, which were significantly lower than for those in the pDCs^low^/Tregs^low^ group (74.9%, 56.8%, and 50.8%, respectively) (Fig. 3a–c). Similarly, the 1-, 3-, and 5-year cumulative recurrence rates for patients in the pDCs^high^/Tregs^high^ group (42.4%, 67.6%, and 73.0%, respectively) were significantly higher than for those in the pDCs^low^/Tregs^low^ group (26.3%, 35.8%, and 43.5%, respectively).

## Discussion

Immune cell abundance is linked to the prognoses of patients with various cancers [6, 23]. Here, we identified peritumoral pDCs as a novel factor for predicting clinical outcomes in patients with ICC. Specifically, a high abundance of pDCs in tissues surrounding the tumor was associated with a shorter OS and greater chance for recurrence. Therefore, pDCs should be examined in biopsy samples taken from patients undergoing curative resection for ICC to identify those at risk for recurrence and reduced survival.

pDCs are phenotypically distinguishable from other dendritic cells [11] and have been detected by immunohistochemical analyses of frozen samples from many types of cancers [13]. In the present study, we utilized a large clinical cohort for which clinical characteristics were known, enabling an investigation of the association between immunohistopathology and prognosis. Moreover, we identified pDCs by using a highly specific and sensitive monoclonal antibody against BDCA2, a member of the C-type lectin family of transmembrane glycoproteins [13, 24, 25], rather than an anti-CD123 antibody, which labels several other cell types [13]. This enabled us to precisely quantify pDCs and assess their clinical significance. We also identified colocalization of BDCA2 and CD123 on most cell surfaces (Additional file 3: Figure S2), which further suggested that BDCA2 is specific to TA-pDCs in HCC. We found that the infiltration of large numbers of pDCs into peritumoral tissues correlated with tumor size and number, vascular/bile duct invasion, lymphatic metastasis, and TNM stage. More importantly, we found that this infiltration is a predictor of TTR and OS, highlighting the importance of the peritumoral microenvironment in ICC progression.

High rates of recurrence or metastasis result in unsatisfactory long-term survival of ICC patients after curative resection [3]. The peritumoral liver tissues were the major target organ of ICC metastasis or recurrence, which was also be classified as intrahepatic recurrence. This peritumoral liver tissue is also reportedly useful for examining intrahepatic recurrence by assessing mechanisms related to immune responses and inflammation [26, 27]. These findings support the “seed and soil” hypothesis of Paget, in which the “seeds” of metastasis can only grow in favorable soil, which includes the target microenvironment [28, 29]. The rate-limiting steps of metastasis or recurrence are tumor cell survival, extravasation, and establishment in the target organ. Consistent with the importance of this microenvironment, we found that the presence of peritumoral pDCs was an independent predicator of ICC prognosis, whereas the presence of intratumor PDCs was not. Together, this demonstrates the prognostic value of the peritumoral microenvironment in metastasizing target organs.

As peri/intratumoral dendritic cells can present tumor-associated antigens to naive T cells, they are thought to trigger an antitumor immune response [30]. Although pDCs synthesize interferon, they do not produce enough to kill cancerous cells due to a lack of appropriate stimuli in the tumor microenvironment or to active suppression [13]. Nevertheless, pDCs stimulate the differentiation and expansion of Tregs in vitro *and* induce Tregs in the periphery in vivo [31, 32]. pDCs have been reported to be capable of directly activating mature Tregs through expression of the enzyme indoleamine 2,3-dioxygenase (IDO). This activation is MHC restricted, requires an intact amino acid-responsive GCN2 pathway in the Tregs, and can be prevented by CTLA4 blockade [33, 34]. Some other reports have also suggested that ICOSL is expressed by pDCs and can interact with ICOS expressed on naïve CD4+ T cells to induce their differentiation into IL-10-producing Tregs [35, 36]. In addition, impaired IFN-α production by pDCs was also shown to be involved in promoting regulatory T cell expansion [37]. However, tumor-infiltrating Tregs may contribute to immune tolerance, as they are associated with the invasiveness and prognosis of most cancer types [6, 38]. Consistent with this, the numbers of pDCs and Tregs in tissues surrounding ICC tumors were correlated with each other and with patient prognosis. By contrast, the accumulation of pDCs within the tumors was not significantly correlated with the numbers of Tregs or prognosis, suggesting that the immunotolerant peritumoral microenvironment is the crucial factor determining ICC outcome.

Although the high recurrence rate of ICC necessitates the ability to predict recurrence, biomarkers for ICC recurrence are lacking. The identification of recurrence predictors could be particularly beneficial to predict and prevent recurrence in early-stage ICC patients who experience relapse unexpectedly just after curative resection. These patients may have a better outcome if recurrence can be predicted early and prevented. Here, we showed that peritumoral pDC levels, may have prognostic ability in identifying these patients. Recurrence is more likely in early-stage or normal CA19-9 ICC patients with high levels of peritumoral pDCs, careful monitoring of such patients is advised.

## Conclusions

We show that peritumoral accumulation of pDCs is a novel predictor of prognosis in patients with ICC undergoing curative resection, which may indicate an immune tolerogenic peritumor microenvironment induced by Foxp3^+^ regulatory T cell infiltration. Future studies investigating therapeutic strategies targeting the anti-immune tolerogenic responses may provide better treatments for patients with ICC.

## Supplementary information

**Additional file 1: Figure S1** Prognostic value of peritumoral pDC in patients with early-stage (TNM stage I) ICC (*n *= 182) or normal carbohydrate antigen 19-9 levels (≤ 36 ng/ml, *n *= 149).

**Additional file 2: Table S1.** Numbers of peritumoral CD3+, CD4+, and CD8+ T cells in ICC cohort and their correlations with pDCs (n=322 for peritumoral tissues).

**Additional file 3: Figure S2** Fluorescence microscopy showed colocalization of BDCA2 and CD123 expression on cell surface.

## Data Availability

The datasets supporting the conclusions of this article are included within the article.

## Abbreviations

pDCs: Plasmacytoid dendritic cells
ICC: Intrahepatic cholangiocarcinoma
TNM: Tumor-node-metastasis
OS: Overall survival
TTR: Time to recurrence
