# Supplementary figures and images for: Peritumoral plasmacytoid dendritic cells predict a poor prognosis for intrahepatic cholangiocarcinoma after curative resection
Source: Cancer Cell Int. 2020 Dec 4;20:582. doi: 10.1186/s12935-020-01676-z (PMC7716503; doi:10.1186/s12935-020-01676-z)

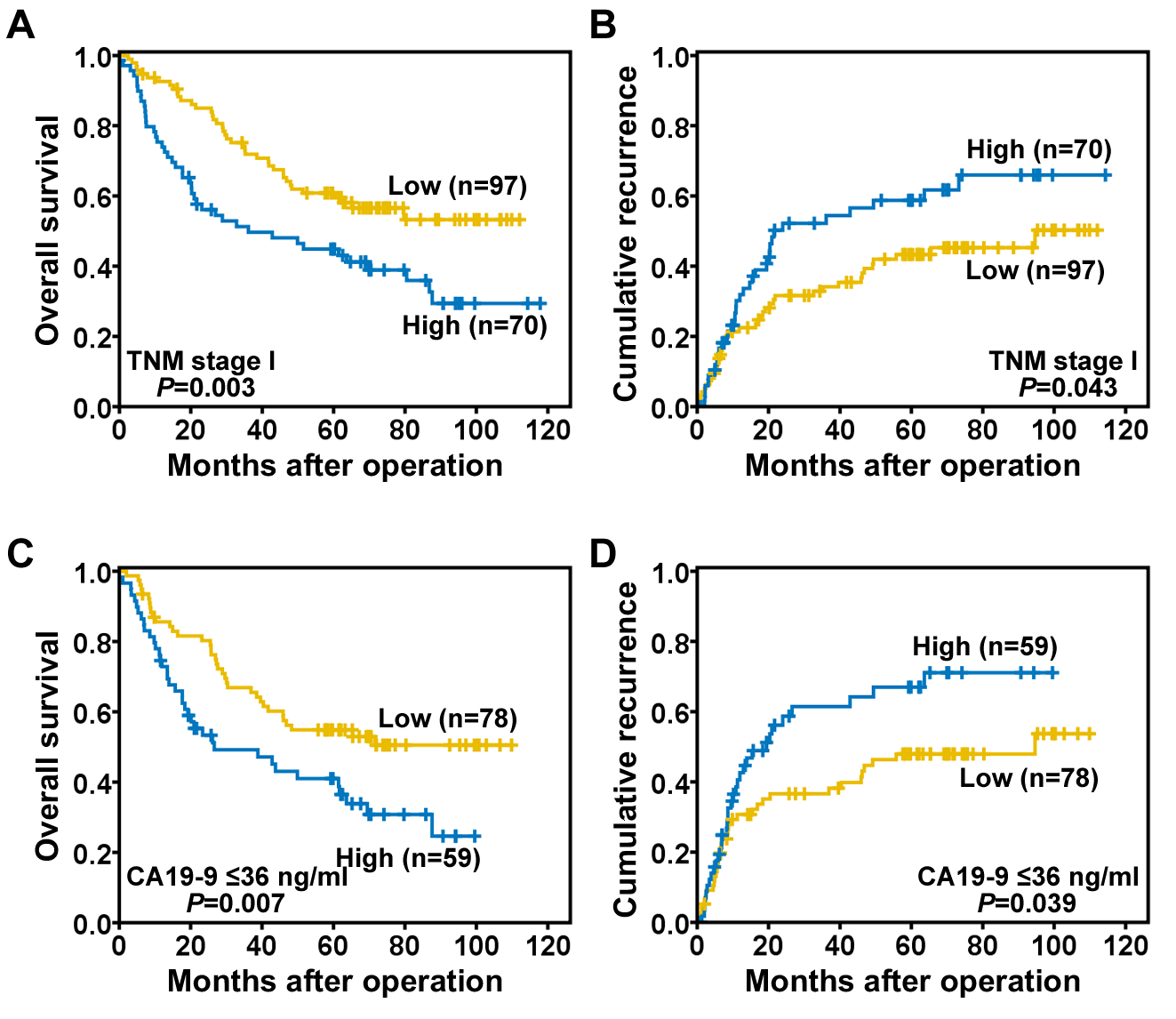

Supplement: Supplementary file 1 — Additional file 1: Figure S1 Prognostic value of peritumoral pDC in patients with early-stage (TNM stage I) ICC (n = 182) or normal carbohydrate antigen 19-9 levels (≤ 36 ng/ml, n = 149). [file 12935_2020_1676_MOESM1_ESM.tif]

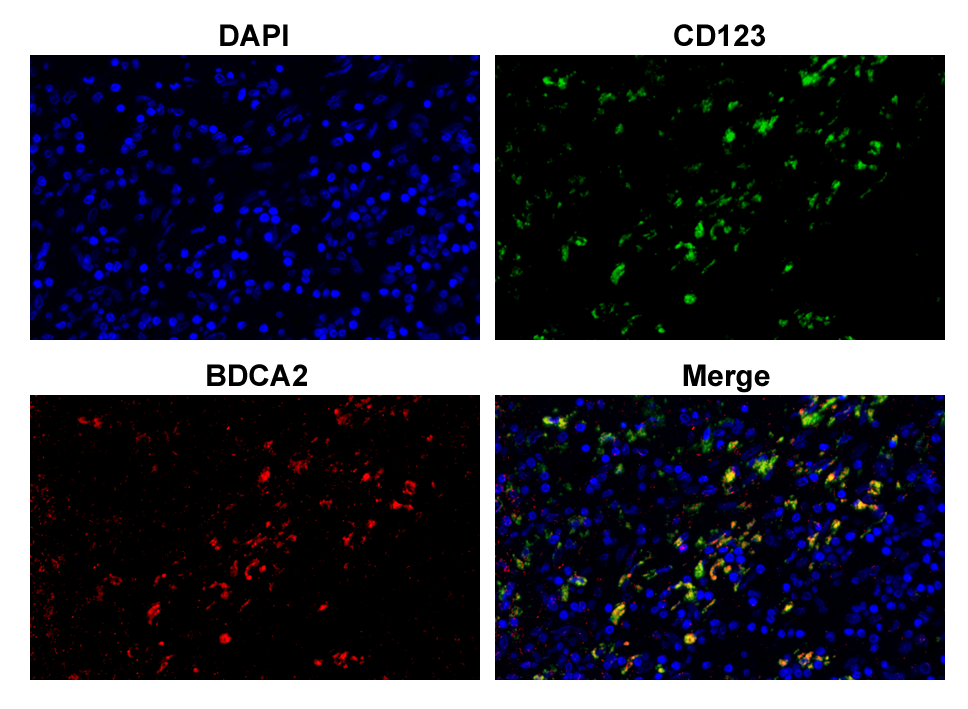

Supplement: Supplementary file 3 — Additional file 3: Figure S2 Fluorescence microscopy showed colocalization of BDCA2 and CD123 expression on cell surface. [file 12935_2020_1676_MOESM3_ESM.tif]
