# Supplementary material for: Peritumoral plasmacytoid dendritic cells predict a poor prognosis for intrahepatic cholangiocarcinoma after curative resection
Source: Cancer Cell Int. 2020 Dec 4;20:582. doi: 10.1186/s12935-020-01676-z (PMC7716503; doi:10.1186/s12935-020-01676-z)
Supplement: Supplementary file 2 — Additional file 2: Table S1. Numbers of peritumoral CD3+, CD4+, and CD8+ T cells in ICC cohort and their correlations with pDCs (n=322 for peritumoral tissues). [file 12935_2020_1676_MOESM2_ESM.doc]

**Additional Table S1.** Numbers of peritumoral CD3+, CD4+, and CD8+ T cells in ICC cohort and their correlations with pDCs (n=322 for peritumoral tissues)**.**

| Variable* | Mean | | | SE | | Range | | Correlations  with pDCs# | |
| --- | --- | --- | --- | --- | --- | --- | --- | --- | --- |
|  | |  |  | |  | |  | |  |
| CD3+  CD4+  CD8+ | 736.2  711.9  246.6 | | | 36.6  25.3  13.4 | | 2-5126  0-3582  0-1325 | | r=0.084, *p*=0.265  r=0.119, *p*=0.086  r=0.032, *p*=0.481 | |

* Number of lymphocytes per 2-mm spot.

# Spearman’s rho coefficients tests.
